# The evolution of three generations of platelet concentrates products: a leap from classical formulations to the era of extracellular vesicles

**DOI:** 10.3389/fbioe.2025.1628565

**Published:** 2025-08-07

**Authors:** Youan Li, Huimin You, Chunhui Ou, Hongyuan Zhu, Biao Cheng, Ju Tian

**Affiliations:** ^1^ Department of Plastic Surgery, ZhongshanCity People’s Hospital, Zhongshan, Guangdong, China; ^2^ Department of Plastic Surgery, Affiliated Hospital of Guangdong Medical University, Zhanjiang, Guangdong, China; ^3^ Department of Plastic Surgery, General Hospital of Southern Theater Command, People's Liberation Army (PLA), Guangzhou, Guangdong, China

**Keywords:** extracellular vesicles, exosomes, classification, regenerative medicine, fibrin scaffold, platelet lysate, cellular therapy platelet concentrates

## Abstract

Platelet concentrates (PCs) have evolved from classical formulations to exosome-based therapies, reflecting a paradigm shift in regenerative medicine. This review analyzes three generations of PCs products, comparing their technological progress, functional differences, and clinical applications. It proposes a novel function-driven classification system that redefines PCs generations based on biological activity rather than chronological development. First-generation PCs, such as platelet-rich plasma (PRP) and platelet-rich growth factor plasma (PRGF), employ centrifugation to concentrate platelets but exhibit limited therapeutic duration due to rapid growth factor depletion and absent fibrin matrices. Second-generation PCs, including platelet-rich fibrin (PRF) and concentrated growth factor (CGF), form natural fibrin networks through low-speed centrifugation, facilitating prolonged cytokine release, though their effectiveness depends heavily on cellular viability. Third-generation PCs represent a paradigm shift by harnessing extracellular vesicles, notably platelet-derived exosomes (PLEXOs). These 30–150 nm vesicles carry growth factors, miRNAs, and lipids, mediating targeted intercellular signaling, immune regulation, and regenerative processes. PLEXOs exhibit greater therapeutic efficacy than previous PCs generations in diverse clinical contexts. Our systematic analysis of PCs evolution and underlying molecular mechanisms addresses three key limitations of extracellular vesicle-based therapies: poor isolation efficiency, regulatory ambiguity, and inconsistent treatment protocols. Critical challenges persist in standardizing extracellular vesicle isolation, scaling production, and validating long-term safety. Future solutions may involve engineered extracellular vesicles, genomic editing, and aptamer-functionalized precision theranostics. The proposed “PRP rapid activation → PRF scaffolding → PLEXOs repair” tri-step therapy demonstrates how intergenerational synergies could advance regenerative medicine with enhanced precision and clinical potential.

## Highlights

• We propose a three-generation classification framework for platelet concentrates (PCs) that reflects their evolving functional roles, from initiating tissue repair to mediating precision therapy through extracellular vesicles.
• Platelet-derived extracellular vesicles emerge as transformative agents for targeted immunomodulation and tissue repair.
• PCs serve as versatile, biomimetic platforms in personalized regenerative medicine, integrating traditional and advanced therapeutic strategies.

## 1 Introduction

Platelet Concentrates (PCs) are a category of high-concentration platelet preparations extracted from patients’ autologous blood through techniques such as centrifugation (Calciolari et al., 2000; Shirbhate and Bajaj, 2022; Stiller et al., 2024; Yang et al., 2025). They are rich in bioactive components, including growth factors, cytokines, and fibrin. Their characteristics of “autologous origin, low immunogenicity, and multiple efficacies” have led to remarkable achievements in fields such as wound repair, tissue regeneration, and aesthetic medicine. The biological effects of PCs stem from their unique composition: growth factors, such as platelet derived growth factor (PDGF), transforming growth factor-β (TGF-β), and vascular endothelial growth factor (VEGF), released upon platelet activation, constitute the core active substances that synergistically regulate cell proliferation, matrix synthesis, and angiogenesis. Leukocyte components (especially neutrophils and monocytes) endow them with anti-inflammatory and immunomodulatory functions. The fibrin scaffold not only serves as a carrier for growth factors but also promotes cell adhesion and three-dimensional migration by mimicking the natural extracellular matrix (ECM) structure. Despite the widespread clinical use of PCs, no consensus exists regarding their generational classification (Calciolari et al., 2000; Shirbhate and Bajaj, 2022; Stiller et al., 2024; Yang et al., 2025), as current models overlook key determinants of therapeutic efficacy, including platelet-derived subcellular components such as exosomes.

This review systematically examines how to classify and enhance the therapeutic potential of PCs to meet clinical demands. We analyzed the developmental trajectory of three PCs generations, detailing their technological advancements, functional distinctions, and clinical implementations. Moving beyond historical progression, we propose a novel three-tier classification framework based on functional mechanisms and molecular composition. The review’s primary contribution lies in establishing this generational classification system, which marks a critical transition from basic platelet concentration to targeted bioactive modulation.

## 2 Discussion

### 2.1 Various concepts and forms of PCs

Over the past decades, various concepts and forms of autologous platelet concentrates have emerged, as detailed below (Table 1).

**TABLE 1:** Comprehensive Table of PCs.

Type	Preparation	Key Features	Applications
PRP (Zhang et al., 2025; Gruber, 2000)	Centrifugation (high-speed); anticoagulants required	High platelet concentration (>1000 × 10^9^/L); rapid GF release (decays in 7 days)	wound healing, orthopedics
PRGF (Anitua et al., 2023; Okada et al., 2016)	Centrifugation (460 g, 8 min); autologous activation	Reduced immunogenicity; no fibrin scaffold	Soft tissue repair, dentistry
PRF (Fursel et al., 2021)	Low-speed centrifugation; no anticoagulants	Dense fibrin network; sustained GF release (14–21 days)	Superficial tissue repair
A-PRF (Farshidfar et al., 2000)	Centrifugation (1300–1500 rpm)	Homogeneous platelet distribution; enhanced regeneration	Chronic wounds, periodontal therapy
i-PRF (Shirbhate and Bajaj, 2022)	Low-temperature centrifugation	Injectable; retains fluidity and bioactivity	Deep-tissue injuries, joint therapy
T-PRF (Silva et al., 2016)	Titanium-modified PRF preparation	Titanium-binding capacity; improved osseointegration	Dental/orthopedic implants
CGF (Chmielewski et al., 2024)	Variable-speed centrifugation (2400–2700 rpm)	High CD34^+^ stem cell content; loose fibrin structure	Angiogenesis, soft tissue regeneration
PL (Lunardon et al., 2024; Bordin et al., 2022)	Freeze-thaw/sonication of platelets	Cell-free; high GF/exosome concentration	Cell culture, tissue engineering
PMVs (Guo et al., 2024)	Released during platelet activation	100–1000 nm; carry CD41/CD62P, cytokines	Intercellular signaling, thrombosis studies
PLEXOs (Wei et al., 2022)	Exocytosis from platelets	30–150 nm; CD63/CD81+; miRNA/protein cargo	Neuroregeneration, immune modulation

#### 2.1.1 platelet-rich plasma (PRP) and plasma rich in growth factors (PRGF)

PRP, first developed in the 1970s, is prepared by centrifuging whole blood to achieve platelet concentrations exceeding 1000 × 10^9^/L, compared to 150–450 × 10^9^/L in whole blood (Zhang et al., 2025; Gruber, 2000). This concentrate rapidly releases growth factors such as PDGF and VEGF, but its therapeutic potential is constrained by transient bioavailability (<7 days) and the absence of a fibrin scaffold. The later introduction of PRGF in the 1990s addressed PRP’s thrombin dependency by utilizing endogenous coagulation pathways for platelet activation (Anitua et al., 2023; Okada et al., 2016). Although PRGF demonstrates improved biocompatibility, inconsistent growth factor release profiles and lack of structural support still limit its utility in tissue regeneration. Both formulations continue to serve as mainstream therapies in orthopedic and sports medicine applications for acute injuries.

#### 2.1.2 Platelet-rich fibrin (PRF) and its advanced derivatives

PRF, developed by Choukroun in the early 2000s, constitutes a structural improvement upon PRP (Fursel et al., 2021). Its preparation through low-speed centrifugation yields a dense fibrin gel that facilitates sustained growth factor release over 14–21 days, though its low mechanical strength restricts application to superficial tissues. Subsequent modifications such as Advanced PRF (A-PRF), centrifuged at 1300–1500 rpm, improve platelet distribution for superior regenerative outcomes (Chmielewski et al., 2024), whereas injectable PRF (i-PRF), introduced in 2013, maintains fluidity for deep tissue administration (Farshidfar et al., 2000). Titanium-enriched PRF (T-PRF), developed in 2014, enhances implant osseointegration through titanium interactions (Shirbhate and Bajaj, 2022), and horizontal centrifugation in Horizontal Platelet-Rich Fibrin (H-PRF) prolongs growth factor retention (Qiu et al., 2023).

Later innovations like lyophilized PRP (Ly-PRP) and lyophilized PRF (Ly-PRF) employ freeze-drying for extended preservation (Silva et al., 2016; Ngah et al., 2021; Anitua et al., 2025), while albumin-bound PRF (Alb-PRF, 2015–2020) incorporates albumin to bolster scaffold stability and growth factor protection (Fujioka-Kobayashi et al., 2021). These developments collectively overcome PRF’s mechanical and storage constraints while broadening its clinical applications.

#### 2.1.3 Concentrated growth factor (CGF)

CGF, introduced in 2006, utilizes variable-speed centrifugation (2400–2700 rpm) to produce a fibrin matrix with greater CD34^+^ stem cell and leukocyte content than PRF (Xiao et al., 2024). The resulting scaffold exhibits elevated growth factor concentrations and a porous fibrin architecture, albeit with reduced mechanical stability for large bone defects. CGF combines the bioactivity of PRP with the sustained release profile of PRF, demonstrating particular efficacy in soft tissue regeneration and periodontal therapies.

#### 2.1.4 Platelet lysate (PL)

Research on PL began comparatively late, with the first studies appearing in the late 1990s. PL consists of platelet components released through physical disruption (e.g., freeze-thaw cycles or sonication) or chemical lysis (e.g., hypotonic treatment), which ruptures cell membranes while preserving cytoplasmic contents, organelle fragments, and platelet-derived extracellular vesicles (p-EVs). Unlike standard PCs, PL contains higher growth factor concentrations and exerts biological effects without requiring intact platelets. This cell-free preparation functions as a bioactive supplement with broad applications in tissue engineering and cell culture (Lunardon et al., 2024; Bordin et al., 2022).

#### 2.1.5 Platelet-derived extracellular vesicles(p-EVs), Platelet microparticles, and Platelet exosomes

Extracellular vesicles (EVs), including exosomes and microparticles, are membrane-bound structures released by cells. Although exosomes and microparticles differ in biogenesis, physical properties, and biological functions, both mediate intercellular communication by transporting molecular cargo. This conserved signaling capability enables EVs to modulate various physiological and pathological processes. Research on platelet-derived microparticles and exosomes has gained significant attention in recent years. Although EVs were first identified in the 1980s, detailed investigations of p-EVs only commenced in the early 2000s. Advances in isolation techniques, such as ultracentrifugation and nanoparticle tracking analysis, have progressively enhanced both fundamental understanding and practical applications of these vesicles (Guo et al., 2024; Yadav et al., 2023; Wei et al., 2022).

Platelet microparticles are small vesicles shed from the cell membrane during platelet activation or apoptosis, typically with diameters ranging from 100 to 1000 nm. They are formed by the encapsulation of cytoplasmic components within the platelet membrane and retain partial platelet membrane proteins like CD41 and CD62P. on their surface. These microparticles contain various bioactive molecules, including growth factors, cytokines, lipids, and nucleic acids. They can act as carriers for intercellular communication, transmitting platelet information to other cells and thereby regulating cellular functions and behaviors (Guo et al., 2024).

Platelet exosomes represent a smaller EV population (30–150 nm) released via exocytosis, distinguished by their endosomal origin and surface markers including CD63, CD81.These vesicles carry abundant nucleic acids (mRNA, miRNA), proteins, and lipids that contribute to intercellular signaling, immune modulation, and tissue regeneration processes (Wei et al., 2022).

### 2.2 Previous traditional classifications, generations, and controversies surrounding PCs

PCs products are referred to by multiple terms, such as platelet-derived biomaterials, wound healing factors, enriched platelet products, and bio-products. This terminological variation implies potential differences in composition and therapeutic effects on tissue regeneration. Recent studies (Dohan et al., 2009; DeLong et al., 2012; Mishra et al., 2012; Mautner et al., 2015; Magalon et al., 2016; Lana et al., 2017; Kawase and Tanaka, 2017; Harrison et al., 2018; Chu et al., 2019; Kon et al., 2020) have introduced classification systems (Table 2) that differentiate PCs products by preparation methods, biological characteristics, and platelet concentrations. The American Academy of Orthopaedic Surgeons established minimal reporting criteria for PRP clinical trials in 2018, requiring documentation of 23 parameters to enhance study reproducibility (Harrison et al., 2018). *Platelets* (Print ISSN: 0953-7104) later mandated 11 essential descriptors for PRP-related manuscripts in 2020 (Harrison and Alsousou, 2020), reinforcing the need for standardized characterization. These developments highlight the critical role of classification systems in ensuring methodological consistency across preclinical and clinical investigations. Sharun (Sharun and Pawde, 2020) further advocated for category-specific reporting standards (*in vitro*, *in vivo*, clinical) to precisely define PRP formulations and preparation protocols.

**TABLE 2:** Classifications of PCs.

Classification	Advantage	Disadvantage	Clinical practicability
Dohan classification, 2009 (Dohan et al., 2009)	Simple and practical,the absolute number of Platelets, and activation methods	Didn’t contain the effects of red blood cells, the absolute number of Platelets, and activation methods	Yes,Classification for PRP and PRF only
PAW classification, 2012 (DeLong et al., 2012)	Contains the absolute number of Platelets, leukocyte concentration and activation method	Didn’t contain the effects of red blood cells, platelet recovery of PRP preparation	Yes,Classification for PRP only
Mishra classification, 2012 (Mishra et al., 2012)	Contains the concentration of platelet, leukocyte and activation method blood cells, platelet recovery of PRP preparation	Didn’t contain the effects of red blood cell, platelet recovery of PRP preparation	Yes,Classification for PRP only
PLRA classification, 2015 (Mautner et al., 2015)	Contains the concentration of platelet, leukocyte and activation method rate of PRP preparation	Didn’t contain platelet recovery ate of PRP preparation	Yes,Classification for PRP only
DEPA classification, 2016 (Magalon et al., 2016)	Contains the concentration of platelet, leukocyte, red blood cells, activation method and platelet recovery	The “ABCD” scoring does not consider the dose or the quality of platelets	Yes,Classification for PRP only
MMARSPILL classi- fication,2017 (Lana et al., 2017)	Contains the concentration of platelet, leukocyte, red blood cells, activation method and preparation process, Image guidance	The classification of platelet activation is too singular	NO,Classification for PRP only Fewer clinical applications
Kawase’s classification (Kawase and Tanaka, 2017)	Contains generic, name principle of preparation and specific materials or procedures, subfamily, brand name, vender	Didn’t contain specific concentrations of components such as platelets, leukocyte, red blood cells	Yes,Classification for PRP and PRF,PRGF,CGF
ISTH classification 2018 (Harrison et al., 2018)	Contains the concentration of platelet, leukocyte, red blood cells, activation and platelet rupture and preparation process	Relatively complex	Yes,Classification for PRP and PRF Fewer clinical applications
Gutierrez’s classifi cation,2019 (Chu et al., 2019)	Contains the concentration of platelet, leukocyte, red blood cells, activation method, and platelet rupture, fibrin, plasma,cell debri, fresh or not	Relatively complex	NO, Classification for PRP; Fewer clinical applications
Kon’s classification, 2020 (Kon et al., 2020)	Platelet composition, purity and activation	Lack of leukocyte, and red blood cell concentrations	NO, Classification for PRP only Fewer clinical applications

Current classification systems categorize PCs into generations as follows: First-Generation (PRP/PRGF) and Second-Generation (PRF/CGF), though some studies classify CGF separately as Third-Generation (Li and Wang, 2024). Third-Generation products comprise A-PRF, i-PRF, T-PRF, H-PRF, Ly-PRF, and Alb-PRF (Table 3). This generational progression reflects three key developments: the shift from anticoagulant-containing to anticoagulant-free preparations, the replacement of chemical anticoagulation in PRP with physical activation in PRF/T-PRF to minimize immunological risks, and structural refinements such as low-speed centrifugation in PRF/A-PRF for natural scaffold formation and variable-speed centrifugation in CGF to improve sustained-release properties. Functional specialization has further diversified these products, with i-PRF designed for injectable applications, Ly-PCs solving preservation difficulties, and Alb-PRF offering enhanced stability.

**TABLE 3:** Previous traditional generations, and limitations of PCs.

Comparison Dimension	First-generation APC (PRP/PRGF)	Second-generation APC (PRF/CGF)	Third-generation APC (A-PRF/i-PRF/T-PRF/H-PRF)
Preparation Principle	Two-step centrifugation (Anticoagulants + Activators)	Single-step centrifugation (No anticoagulants)	Optimized centrifugation parameters (Low-speed/short-time) or morphological modifications
Platelet Concentration	4–8× enrichment (∼1,000 × 10^9^/L)	3–5× enrichment (∼800 × 10^9^/L)	3–8× enrichment (Varies with centrifugation conditions)
Leukocyte Content	None or minimal (Requires additional supplementation)	High concentration (Similar to whole blood)	Neutrophil-enriched distribution
Red Blood Cell Residue	Contains RBCs (May trigger inflammation)	RBC-free	RBC-free
Fibrin Architecture	Porous network (Requires exogenous thrombin activation)	Dense 3D network (Natural coagulation mechanism)	Adjustable density/porosity(A-PRF/i-PRF/T-PRF variants)
Growth Factor Release	Rapid release (Peak at 5–10 min)	Sustained release (7–14 days)	Controlled release kinetics (Dependent on structural optimization)
Activation Method	Exogenous activation (CaCl_2_, thrombin)	Endogenous activation (Autologous thrombin)	Endogenous activation (No exogenous additives)
Representative Technologies	PRP, PRGF	PRF, CGF	A-PRF, i-PRF, T-PRF, H-PRF
Limitations	Short duration, immunogenicity, thrombotic risk	Mechanical weakness, technical variability	Equipment dependency, standardization gaps

### 2.3 The limitations of traditional classifications

The current classification and generational categorization of PCs have facilitated their clinical application to some extent. However, several significant limitations remain, as outlined below:

#### 2.3.1 Ambiguity in functional differentiation

The current classification system for PCs primarily focuses on preparation techniques and compositional profiles but fails to adequately capture their functional heterogeneity. While PRP, PRF, A-PRF, and CGF differ in preparation methods and structural properties, existing taxonomies poorly characterize their therapeutic differences, particularly regarding physical states (liquid versus solid matrices) and growth factor release kinetics (immediate versus sustained).

Significant variation in cellular composition, especially leukocyte and erythrocyte concentrations, complicates standardization efforts. Leukocytes—mainly neutrophils and monocytes—exhibit context-dependent therapeutic effects, offering benefits in some clinical scenarios while potentially causing harm in others. In infected or inflammatory environments, they contribute to antimicrobial defense and immunomodulation through cytokine release, whereas under sterile conditions, leukocyte-derived reactive oxygen species and pro-inflammatory mediators may hinder tissue regeneration by amplifying oxidative stress. This biological duality highlights the need for strategic leukocyte management across PC formulations. PRP often reduces leukocyte content to minimize inflammation, whereas PRF and CGF retain leukocytes to prolong cytokine activity. PLEXOs represent a further refinement, leveraging platelet-derived exosomes (30–150 nm) to deliver targeted molecular payloads while avoiding leukocyte-related complications. Thus, optimal leukocyte incorporation must be carefully matched to the specific pathophysiology, emphasizing the importance of precision medicine in PC selection.

#### 2.3.2 Overlooking the potential of PL, Platelet microparticles, and Platelet exosomes

The failure to recognize the value of PL, platelet microparticles, and platelet exosomes overlooks a critical avenue for innovation in platelet-based therapeutics. These platelet-derived components contain diverse bioactive molecules—growth factors, signaling molecules, nucleic acids, and proteins—that regulate cell proliferation, immune modulation, tissue repair, targeted drug delivery, and diagnostic applications. Current research limitations and technological barriers have prevented their full characterization and utilization, obscuring their translational potential. This gap hinders advances in platelet product development for regenerative medicine, precision medicine, and multimodal disease management.

Inconsistent preparation protocols further complicate the field. While traditional classification systems outline platelet product manufacturing methods, procedural variations and the absence of standardized protocols yield substantial batch-to-batch and intermanufacturer heterogeneity in product quality and biological activity. Such variability compromises clinical performance while introducing unnecessary therapeutic uncertainty.

#### 2.3.3 Inadequate guidance for clinical applications

Current generation classification systems offer limited practical guidance for clinical decision-making. These frameworks fail to address critical questions regarding optimal product selection, administration protocols, or expected therapeutic outcomes for specific diseases or anatomical sites. Without such evidence-based recommendations, clinicians struggle to maximize the therapeutic potential of platelet-derived therapies.

The heterogeneity in PCs terminology and generational classification mirrors advancements in production methods—including anticoagulant-free processing and low-speed centrifugation—and diverse functional objectives like sustained release or structural support. Yet existing classification systems exhibit three key shortcomings: overlapping functional profiles across generations, insufficient exploitation of platelet lysates and derivatives, and variable efficacy due to inconsistent manufacturing standards. Furthermore, they provide no systematic guidance for clinical implementation. Establishing standardized nomenclature, production protocols, and functional validation would significantly enhance their utility in regenerative medicine.

### 2.4 A new generational framework: three-generation classification based on functional drivers and molecular mechanisms

**FIGURE 1:**
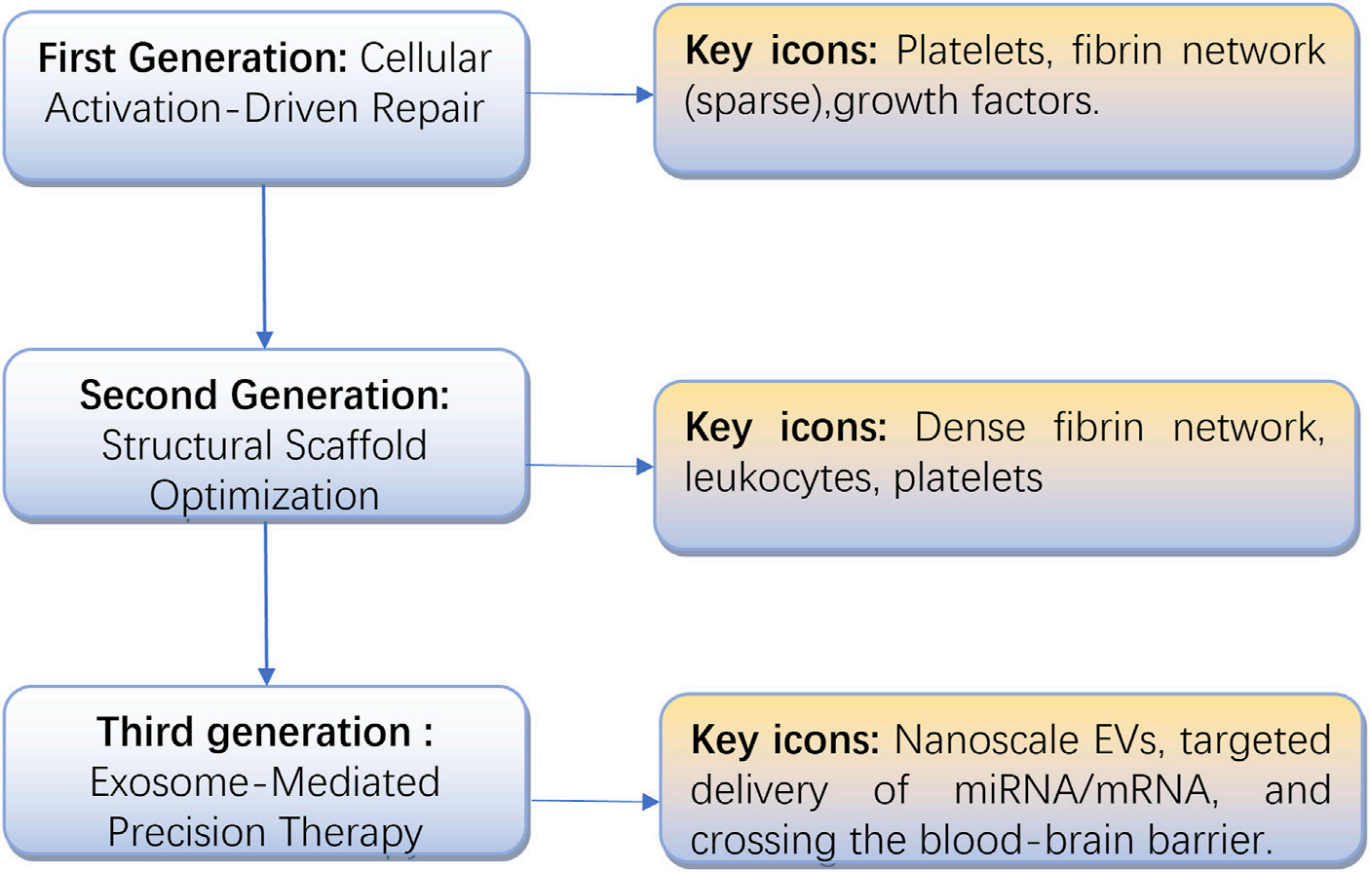
The evolution of platelet concentrates, from cellular activation to exosome-based precision therapy.

Platelet-derived products have become fundamental in regenerative medicine, evolving from basic platelet concentrates like PRP and PRGF—prepared through centrifugation—to advanced fibrin-based scaffolds such as PRF and CGF. These innovations have now progressed beyond cellular carriers, embracing cell-free approaches that harness extracellular vesicles, particularly platelet-derived exosomes (as shown in Figure 1).

This progression reflects not only technological advancements, from simple growth factor release to precise molecular signaling control, but also a broader shift in regenerative medicine from empirical methods to mechanism-based strategies. To assess these developments, we systematically reviewed the characteristics and clinical performance of various platelet-derived products, identifying key challenges in standardization and proposing potential solutions. Building on these findings, we present a new three-generation classification system based on functional mechanisms and molecular drivers, aiming to support the translation of exosome-focused regenerative therapies (Table 4).

**TABLE 4:** A new generational framework: Three-generation classification based on functional drivers and molecular mechanisms.

Category	Preparation principle	Key components	Mechanism of Action	Advantages	Disadvantages	Standardization challenges
First-Generation: PRP	Gradient centrifugation separates PRP; requires anticoagulants (e.g., CaCl_2_) and activators (e.g., thrombin)	High platelet concentration (1,000 × 10^9^/L), PDGF, TGF-β, VEGF, leukocytes, erythrocytes	Rapid growth factor release (half-life <24 h); activates osteoblasts/fibroblasts via paracrine signaling	Simple preparation, low cost, autologous	Short duration of action, batch variability, EDTA-related tissue damage, thrombotic risks with PRP.	Centrifugation parameters, leukocyte contamination, and growth factor quantification
First-Generation: PRGF	Selective activation of platelets (anticoagulant-free)	Low platelet count, high growth factor concentration, fibrin-free matrix	Rapid growth factor release (similar to PRP); no anticoagulant residue	No EDTA interference, immediate usability	Short GF duration, lacks structural support	Component variability, quality control inconsistencies
Second-Generation: PRF	Low-speed centrifugation (≤1500 rpm) in non-anticoagulant tubes forms a porous fibrin network	Fibrin scaffold (3D network), platelets, leukocytes, growth factors (PDGF-BB↑)	Sustained growth factor release (14–21 days); modulates inflammation via leukocytes	Natural fibrin architecture, leukocyte-mediated antimicrobial effects, reduced immunogenicity	Weak mechanical strength, unsuitable for large bone defects, manual preparation variability	Fibrin structure standardization, batch reproducibility
Second-Generation: CGF	Ultracentrifugation (2400–2700 rpm) concentrates platelet microparticles and growth factors	Dense fibrin matrix, elevate, rich in CD34 + stem cells	Enhanced growth factor retention, angiogenic potential, and controlled degradation kinetics	Higher growth factor density, tailored degradation rates	Complex preparation protocol, cardiovascular thrombosis risks	Centrifugation speed standardization, scalability
Third-Generation: EVs, PLEXOs	Freeze-thaw or enzymatic lysis of platelets releases extracellular vesicles (exosomes)	Platelet-derived exosomes (30–150 nm), miRNAs (miR-21, miR-126), proteins, lipids	Targeted signaling via exosomal cargo (e.g., RUNX2 for osteogenesis); bypasses thrombogenic risks	Non-cellular, immune-evasive, crosses blood-brain barrier, scalable mRNA/drug loading	Low exosome yield (<30%), purification complexity, regulatory hurdles (EMA Category III)	Exosome isolation standards (CD63/CD81 markers), clinical translation gaps
​Third-Generation: PL	Freeze-thaw/ultrasonic lysis of platelets	High growth factor/exosome/cytokine concentration, cell-free debris	Exosome-mediated signaling; synergistic growth factor activity	Non-immunogenic, scalable industrial production	Harsh storage requirements, active component degradation	Freeze-thaw protocol standardization, transportation costs
Emerging Derivatives						
I-PRF	Short-spin centrifugation (700 rpm, 3 min) retains high leukocyte concentration (20× PRP)	Leukocyte-rich fibrin matrix, platelet cytokines, growth factors	Immediate cytokine release; supports angiogenesis and soft tissue integration	High leukocyte content, injectability, minimal processing time	Limited growth factor longevity, undefined fibrin structure	Standardization of spin parameters, clinical endpoint validation
T-PRF	Centrifugation in titanium tubes enhances fibrin cross-linking and mechanical stability	Titanium-modified fibrin network, sustained growth factor release (up to 14 days)	Improved structural integrity; supports osseointegration and long-term tissue remodeling	Superior mechanical properties, extended growth factor delivery	Higher cost, titanium residue concerns	Titanium surface chemistry optimization
H-PRF	Horizontal centrifugation (700–1200 rpm)	Bilayer fibrin matrix (dense + porous)	Prolonged GF release (>28 days) via CD34^+^ cell modulation	Ultra-long GF action, high vascularization potential, reduced thrombosis risk	Equipment dependency, technical sensitivity	Centrifugation protocol standardization
A-PRF	Extended low-speed centrifugation (1300–1500 rpm)	Dense fibrin network High cellularity	Sustained GF release (14–28 days) via leukocyte-mediated anti-inflammation	Superior bone/soft tissue regeneration, cost-effective	Manual variability, inconsistent fibrin structure	Centrifugation duration/protocol validation

#### 2.4.1 First generation

Classic PCs (PRP/PRGF), presented as liquid bioactive solutions. Centered on platelet aggregation effects, these concentrates contain high concentrations of platelets, red blood cells, white blood cells, and plasma proteins. Their mechanism relies primarily on platelet aggregation, which induces the transient release of growth factors such as PDGF and TGF-β from α-granules upon activation with calcium ions or collagen. This process stimulates osteoblast and fibroblast activity, though the therapeutic effects remain short-lived.

#### 2.4.2 Second generation

The transition from first-generation PCs (PRP/PRGF) to second-generation fibrin-based scaffolds (PRF/CGF) reflects a paradigm shift in regenerative therapeutics. Unlike first-generation products, which relied on anticoagulant protocols to enrich platelets at the cellular level, second-generation approaches optimized structural properties by developing endogenous fibrin matrices. PRF preserves fibrin networks at low temperatures to facilitate prolonged growth factor release, while CGF utilizes variable-speed centrifugation to concentrate platelet microparticles and modulate growth factor profiles. This shift from liquid suspensions to three-dimensional bioactive scaffolds redefined therapeutic strategies, replacing transient growth factor delivery with microenvironment-mimicking tissue engineering.

Although second-generation technologies improved functionality, their dependence on cellular integrity led to advanced derivatives such as A-PRF, i-PRF, and T-PRF. These variants modify fibrin architecture—exemplified by H-PRF’s horizontal centrifugation—or enhance stability through methods like albumin incorporation in Alb-PRF, while maintaining the focus on structural optimization. This progression highlights a key trend in regenerative medicine: advancing from cellular concentration toward biomaterial-driven, spatiotemporally controlled tissue repair, a principle further refined by third-generation cell-free exosome therapies.

#### 2.4.3 Third generation:extracellular vesicle carriers, exosome-oriented products

EVs and exosomes provide unique benefits as cell-free systems that avoid complications from platelet aggregation. p-EVs therapeutics, particularly PLEXOs, represent a transformative development in regenerative medicine compared to conventional approaches (Wan et al., 2024; Gardin et al., 2022; Wei et al., 2024; Gupta et al., 2025; Li et al., 2024). These platforms, ranging from fibrin-based scaffolds like PRF and CGF to engineered exosomes, enable the targeted delivery of physiologically balanced growth factors, miRNAs, and signaling molecules with precise spatiotemporal control. Their autologous origin guarantees immune compatibility, while exosomes exploit intrinsic targeting mechanisms to efficiently cross biological barriers, including the blood-brain barrier. Although regulatory classification and cost-effectiveness remain unresolved, platelet-derived systems mark a paradigm shift from empirical cell transplantation to controlled, patient-specific tissue regeneration. By integrating natural healing processes with bioengineering precision, these therapeutics are reshaping regenerative therapy strategies.

This evolution expands the scope of regenerative medicine, yet each generation of platelet-derived therapeutics faces distinct standardization hurdles. The first generation contends with concentration variability due to centrifuge parameters, which closed systems can minimize. The second generation overcomes fibrin structure inconsistencies caused by manual preparation through automated standardization. The third generation resolves low exosome purity by implementing advanced extraction and purification methods, setting critical benchmarks for clinical-grade exosome therapy.

### 2.5 Future prospects

Future research on PCs should prioritize refining the three-generation classification framework, progressing from classic concentrates to fibrin scaffolds and exosome carriers, while optimizing functionality, delivery systems, safety profiles, and standardization protocols. This evolution marks a transition from passive therapeutic use to actively regulated regenerative medicine strategies. Standardizing preparation methods—including centrifugation parameters and activation techniques—will enhance batch consistency, supported by thorough quantification of bioactive components. Preclinical validation must integrate *in vitro* functional assays, relevant animal models, and well-controlled clinical trials with placebo comparators and therapeutic benchmarks, rigorously employing blinding and randomization. These measures will clarify relationships between product attributes and clinical outcomes, overcoming existing reproducibility and evidence gaps that limit translational progress.

Specifically, for each generation of PCs, improvements can be pursued in the following directions.

#### 2.5.1 First generation: intelligent upgrades of classic concentrates

While first-generation PCs (PRP/PRGF) remain clinically valuable for their rapid growth factor release, their transient therapeutic effects limit long-term efficacy. To address this, emerging nano-engineering strategies focus on sustained delivery systems.

#### 2.5.2 Second generation: functional reshaping of fibrin scaffolds

Although PRF and CGF achieve the sustained release of growth factors through structural optimization, their dependence on the integrity of the cytoskeleton limits their application scenarios. Current research focuses on structural optimization using cross-linking agents to enhance scaffold stability against enzymatic degradation (Karimi et al., 2022). Particularly promising is the integration with gelatin methacryloyl (GelMA), a photo-crosslinkable hydrogel that enables 3D printing of porous scaffolds (80%–90% porosity) (Liang et al., 2023). This combination approach has demonstrated particular potential for complex wound healing applications, such as diabetic foot ulcers, by providing both structural support and controlled bioactive release.

#### 2.5.3 Third generation: The precision revolution of exosome carriers

PLEXOs show promise as cell-free therapeutic carriers, but their clinical translation requires addressing several key challenges. Dual-targeted exosomes (CD47 + RGD) overcome critical limitations in targeted delivery and immune evasion by simultaneously preventing macrophage phagocytosis through CD47’s “do not eat me” signal and enhancing cellular uptake via RGD-mediated integrin binding (Xie et al., 2023). This dual-targeting strategy increases exosome accumulation at disease sites, facilitating tissue regeneration. CRISPR-associated protein 9 (CRISPR - Cas9) technology further expands therapeutic potential by enabling precise exosomal cargo modifications, such as miR-34a knockout to suppress tumor metastasis (Aslan et al., 2024). The SpyTag/SpyCatcher system permits covalent attachment of functional proteins to exosome surfaces, creating versatile platforms for vaccines, targeted therapies, and diagnostic applications (Zheng et al., 2024). These engineered exosomes can respond to environmental stimuli or enzymatic triggers, advancing precision medicine approaches.

## 3 Results

In summary, a scientific and rational classification system for concentrated platelet generations shifts the focus from simple platelet enrichment to developing tools with multidimensional bioactive regulatory functions. Future advances in this field will likely center on standardizing preparation protocols, advancing composite biomaterials, and optimizing personalized treatment strategies, thereby enhancing precision in regenerative medicine. These developments may also foster intergenerational collaboration, creating a more integrated regenerative medicine ecosystem. A multigenerational product combination strategy—such as employing PRP for rapid activation, PRF scaffolds for filling, and PLEXOs for sustained repair—could synergize therapeutic effects across product generations. Such an approach may provide innovative solutions for complex clinical challenges.

## 4 Conclusion

This review proposes a function-driven, three-tiered classification system for platelet concentrates, redefining therapeutic approaches that extend beyond simple platelet enrichment. The framework distinguishes first-generation PRP/PRGF (rapid activation), second-generation PRF/CGF (sustained structural support), and third-generation PLEXOs (precise EVs signaling), highlighting a critical transition from empirical platelet activation to engineered molecular delivery. Third-generation therapies mark a fundamental shift by utilizing EVs as targeted bioactive carriers for immunomodulation and tissue regeneration, though challenges remain in scaling production, standardizing mechanisms, and translating findings to clinical practice.

## References

[B1] Anitua E. Fuente M. Alkhraisat M. H. (2025). Long term stability of preservative-free and lyophilized PRGF eye drops stored at different temperature conditions: *in vitro* comparative study. CRYOBIOLOGY 119, 105214. 10.1016/j.cryobiol.2025.105214 39956350

[B2] Anitua E. Muruzabal F. de la Fuente M. Del Olmo-Aguado S. Alkhraisat M. H. Merayo-Lloves J. (2023). PRGF membrane with Tailored Optical properties preserves the Cytoprotective effect of plasma rich in growth factors: *in vitro* model of Retinal Pigment Epithelial cells. Int. J. Mol. Sci. 24 (13), 11195. 10.3390/ijms241311195 37446374 PMC10342881

[B3] Aslan C. Zolbanin N. M. Faraji F. Jafari R. (2024). Exosomes for CRISPR-Cas9 delivery: the Cutting Edge in Genome editing. Mol. Biotechnol. 66 (11), 3092–3116. 10.1007/s12033-023-00932-7 38012525

[B4] Bordin A. Chirivì M. Pagano F. Milan M. Iuliano M. Scaccia E. (2022). Human platelet lysate-derived extracellular vesicles enhance angiogenesis through miR-126. Cell Prolif. 55 (11), e13312. 10.1111/cpr.13312 35946052 PMC9628251

[B5] Calciolari E. Dourou M. Akcali A. Donos N. (2000). Differences between first- and second-generation autologous platelet concentrates. PERIODONTOL 97 (1), 52–73. 10.1111/prd.12550 PMC11808449 38487938

[B6] Chmielewski M. Pilloni A. Adamska P. (2024). Application of advanced platelet-rich fibrin in oral and Maxillo-Facial Surgery: a systematic review. J. Funct. Biomater. 15 (12), 377. 10.3390/jfb15120377 39728177 PMC11678554

[B7] Chu C. R. Rodeo S. Bhutani N. Goodrich L. R. Huard J. Irrgang J (2019). Optimizing clinical Use of Biologics in Orthopaedic Surgery: consensus recommendations from the 2018 AAOS/NIH U-13 Conference. J. Am. Acad. Orthop. SUR 27 (2), e50–e63. 10.5435/jaaos-d-18-00305 PMC6314629 30300216

[B8] DeLong J. M. Russell R. P. Mazzocca A. D. (2012). Platelet-rich plasma:the PAW classification system. Arthroscopy 28 (7), 998–1009. 10.1016/j.arthro.2012.04.148 22738751

[B9] Dohan E. D. M. Rasmusson L. Albrektsson T. (2009). Classification of platelet con- Centrates: from pure platelet-rich plasma (P-PRP) to leucocyte- and platelet-rich fibrin (L-PRF). Trends Biotechnol. 27 (3), 158–167. 10.1016/j.tibtech.2008.11.009 19187989

[B10] Farshidfar N. Amiri M. A. E Estrin N. Ahmad P. Sculean A. Zhang Y. (2000). Platelet-rich plasma (PRP) versus injectable platelet-rich fibrin (i-PRF): a systematic review across all fields of medicine. PERIODONTOL. 10.1111/prd.12626 PMC13428083 40125556

[B11] Fujioka-Kobayashi M. Schaller B. Mourão CFAB Zhang Y. Sculean A. Miron R. J. (2021). Biological characterization of an injectable platelet-rich fibrin mixture consisting of autologous albumin gel and liquid platelet-rich fibrin (Alb-PRF). PLATELETS. 2021-01-02 32 (1), 74–81. 10.1080/09537104.2020.1717455 31959025

[B12] Fursel K. Oliveira Neto J. Sousa M. Moreira V. Silveira R. (2021). Propriedades da fibrina rica em plaquetas (PRF) aplicada a cirurgia oral - protocolo Choukroun. Choukroun Res. Soc. Dev. 10 (5), e59510515338. 10.33448/rsd-v10i5.15338

[B13] Gardin C. Ferroni L. Leo S. Tremoli E. Zavan B. (2022). Platelet-derived exosomes in Atherosclerosis. Int. J. Mol. Sci. 23 (20), 12546. 10.3390/ijms232012546 36293399 PMC9604238

[B14] Gruber R. (2000). How to explain the beneficial effects of platelet-rich plasma. Periodontol 97 (1), 95–103. 10.1111/prd.12565 PMC11808461 38600634

[B15] Guo J. Cui B. Zheng J. Yu C. Zheng X. Yi L. (2024). Platelet-derived microparticles and their cargos: the past, present and future. Asian J. Pharm. Sci. 19 (2), 100907. 10.1016/j.ajps.2024.100907 38623487 PMC11016590

[B16] Gupta A. K. Wang T. Rapaport J. A. Talukder M. (2025). Therapeutic potential of extracellular vesicles (exosomes) derived from platelet-rich plasma: a Literature review. J. Cosmet. Dermatol 24 (2), e16709. 10.1111/jocd.16709 39618056 PMC11845942

[B17] Harrison P. Alsousou J. (2020). Studies on platelet rich plasma - new editorial policy for“Platelets”. Platelets 3 (3), 281–282. 10.1080/09537104.2020.1729013 32124684

[B18] Harrison P. Alsousou J. Andia I. Burnouf T. Dohan Ehrenfest D. Everts P. (2018). The use of platelets in regenerative medicine and proposal for a new classification system: guidance from the SSC of the ISTH. J. ThrombHaemost 16 (9), 1895–1900. 10.1111/jth.14223 30099839

[B19] Karimi F. Biazar E. Heidari-Keshel S. Pourjabbar B. Khataminezhad M. R. Shirinbakhsh S. (2022). Platelet-rich fibrin (PRF) gel modified by a carbodiimide crosslinker for tissue regeneration. RSC Adv. 12 (21), 13472–13479. 10.1039/d2ra00985d 35527730 PMC9069288

[B20] Kawase T. Tanaka T. (2017). An updated proposal for terminology and classification of platelet-rich fibrin. Regen. Ther. 7, 80–81. 10.1016/j.reth.2017.10.002 30271855 PMC6153447

[B21] Kon E. Di M. B. Delgado D. Cole B. J. Dorotei A. Dragoo J. L. (2020). Platelet-rich plasma for the treatment of knee osteoarthritis: an expert opinion and proposal for a novel classification and coding system. Expert Opin. Biol. Ther. 2020-12-01; 20 (12), 1447–1460. 10.1080/14712598.2020.1798925 32692595

[B22] Lana J. Purita J. Paulus C. Huber S. C. Rodrigues B. L. Rodrigues A. A. (2017). Contributions for classification of platelet rich plasma - proposal of a new classification: MARSPILL. Regen. Med. 12 (5), 565–574. 10.2217/rme-2017-0042 28758836

[B23] Li G. Wang H. (2024). Novel applications of concentrated growth factors in facial Rejuvenation and Plastic Surgery. FACIAL PLAST. Surg. 40 (1), 112–119. 10.1055/a-1987-3459 36423628

[B24] Li X. Guo F. Deng J. Li J. Zhang J. Fu M. (2024). Leukocyte platelet-rich plasma-derived exosomes Restrained macrophages viability and induced apoptosis, NO generation, and M1 polarization. Immun. Inflamm. Dis. 12 (11), e70064. 10.1002/iid3.70064 39545659 PMC11565605

[B25] Liang J. Wang Z. Poot A. A. Grijpma D. W. Dijkstra P. J. Wang R. (2023). Enzymatic post-crosslinking of printed hydrogels of methacrylated gelatin and tyramine-conjugated 8-arm poly(ethylene glycol) to prepare interpenetrating 3D network structures. Int. J. Bioprint 9 (5), 750. 10.18063/ijb.750 37457933 PMC10339421

[B26] Lunardon T. Sumner S. M. Mollabashi M. Darzenta N. Davis E. Naskou M. C. (2024). Growth factor and cytokine characterization of canine platelet lysate with variable leukocyte concentration, plasma content, and heat-sensitive proteins. Front. Vet. Sci. 11, 1408080. 10.3389/fvets.2024.1408080 39071789 PMC11272652

[B27] Magalon J. Chateau A. L. Bertrand B. Louis M. L. Silvestre A. Giraudo L. (2016). DEPA classification: a proposal for standardising PRP use and a retrospective application of available devices. BMJ Open Sport Exerc Med. 2 (1), e000060. 10.1136/bmjsem-2015-000060 PMC5117023 27900152

[B28] Mautner K. Malanga G. A. Smith J. Shiple B. Ibrahim V. Sampson S. (2015). A call for a standard classification system for future biologic research: the rationale for new PRP nomenclature. PM &R J. Inj. Funct. rehabilitation 7 (4 Suppl. l), S53-S59–S9. 10.1016/j.pmrj.2015.02.005 25864661

[B29] Mishra A. Harmon K. Woodall J. Vieira A. (2012). Sports medicine applications of platelet rich plasma. Curr. Pharm. Biotechno 13 (7), 1185–1195. 10.2174/138920112800624283 21740373

[B30] Ngah N. A. Dias G. J. Tong D. C. Mohd Noor S. N. F. Ratnayake J. Cooper P. R. (2021). Lyophilised platelet-rich fibrin: physical and biological Characterisation. Molecules 26 (23), 7131. 10.3390/molecules26237131 34885714 PMC8658988

[B31] Okada H. Takahashi K. Ogura N. Tomoki R. Ito K. Kondoh T. (2016). Plasma rich in growth factors stimulates proliferation, migration, and gene expression associated with bone formation in human dental follicle cells. J. Dent. Sci. 11 (3), 245–252. 10.1016/j.jds.2015.12.001 30894980 PMC6395260

[B32] Qiu Y. Bao S. Wei H. Miron R. J. Bao S. Zhang Y. (2023). Bacterial exclusion and wound healing potential of horizontal platelet-rich fibrin (H-PRF) membranes when compared to 2 commercially available collagen membranes. Clin. ORAL INVEST 27 (8), 4795–4802. 10.1007/s00784-023-05108-w 37318640

[B33] Sharun K. Pawde A. M. (2020). *In vitro* studies using platelet-rich plasma: Minimum reporting requirements. Cell Biol. Int. 44 (12), 2380–2382. 10.1002/cbin.11462 32902883

[B34] Shirbhate U. Bajaj P. (2022). Third-generation platelet concentrates in periodontal regeneration: Gaining Ground in the field of regeneration. Cureus 14 (8), e28072. 10.7759/cureus.28072 36127983 PMC9477433

[B35] Silva L. Huber S. Montalvão S. Bassora F. De Paula E. Annichino-Bizzacchi J. (2016). Platelet activation is not Crucial for platelet-rich plasma (PRP), when used as autologous therapeutic product, and could be lyophilized without. Any Growth Factor Loss Blood. 2016-12-02 128 (22), 2639. 10.1182/blood.v128.22.2639.2639

[B36] Stiller H. L. Perumal N. Manicam C. Trzeciak E. R. Todt J. Jurk K. (2024). First-vs. Second-generation autologous platelet concentrates and their Implications for wound healing: differences in Proteome and Secretome. Bioeng. (Basel) 11 (11), 1171. 10.3390/bioengineering11111171 PMC11591784 39593831

[B37] Wan P. Tan X. Sheng M. Xiang Y. Wang P. Yu M. (2024). Platelet exosome-derived miR-223-3p regulates Pyroptosis in the cell model of Sepsis-Induced acute Renal Injury by targeting mediates NLRP3. Crit. Rev. Immunol. 44 (3), 53–65. 10.1615/CritRevImmunol.2023051651 38421705

[B38] Wei K. Huang H. Liu M. Shi D. Ma X. (2022). Platelet-derived exosomes and Atherothrombosis. Front. Cardiovasc Med. 9, 886132. 10.3389/fcvm.2022.886132 35498048 PMC9051247

[B39] Wei K. Yu L. Li J. Gao J. Chen L. Liu M. (2024). Platelet-derived exosomes regulate endothelial cell inflammation and M1 macrophage polarization in coronary artery thrombosis via modulating miR-34a-5p expression. Sci. Rep. 14 (1), 17429. 10.1038/s41598-024-67654-x 39075107 PMC11286768

[B40] Xiao Q. Chu W. Guo J. Gao J. Yao W. Huang M. (2024). CGF therapy: bridging androgenetic alopecia observations to psoriasis treatment via IL-17 pathway. Stem Cell Res. Ther. 15 (1), 353. 10.1186/s13287-024-03959-y 39380104 PMC11462746

[B41] Xie Y. Sun Y. Liu Y. Zhao J. Liu Q. Xu J (2023). Targeted delivery of RGD-CD146^+^CD271^+^ human Umbilical Cord Mesenchymal stem cell-derived exosomes promotes blood-Spinal Cord barrier repair after Spinal Cord Injury. ACS Nano 17 (18), 18008–18024. 10.1021/acsnano.3c04423 37695238

[B42] Yadav P. Beura S. K. Panigrahi A. R. Bhardwaj T. Giri R. Singh S. K. (2023). Platelet-derived microvesicles activate human platelets via intracellular calcium mediated reactive oxygen species release. Blood Cells Mol. Dis. 98, 102701. 10.1016/j.bcmd.2022.102701 36057195

[B43] Yang M. Deng B. Hao W. Jiang X. Chen Y. Wang M. (2025). Platelet concentrates in diabetic foot ulcers: a comparative review of PRP, PRF, and CGF with case insights. Regen. Ther. 28, 625–632. 10.1016/j.reth.2025.02.005 40166040 PMC11955794

[B44] Zhang Z. Liu P. Xue X. Zhang Z. Wang L. Jiang Y. (2025). The role of platelet-rich plasma in biomedicine: a comprehensive overview. iScience 28 (2), 111705. 10.1016/j.isci.2024.111705 39898035 PMC11787504

[B45] Zheng L. Li J. Li Y. Sun W. Ma L. Qu F. (2024). Empowering exosomes with aptamers for precision Theranostics. Small Methods 5, e2400551. 10.1002/smtd.202400551 38967170

